# Development and Validation of Autophagy-Related Gene Signature and Nomogram for Predicting Survival in Oral Squamous Cell Carcinoma

**DOI:** 10.3389/fonc.2020.558596

**Published:** 2020-10-16

**Authors:** Chen Hou, Hongshi Cai, Yue Zhu, Shuojin Huang, Fan Song, Jinsong Hou

**Affiliations:** ^1^ Department of Oral and Maxillofacial Surgery, Guanghua School of Stomatology, Hospital of Stomatology, Sun Yat-sen University, Guangzhou, China; ^2^ Guangdong Provincial Key Laboratory of Stomatology, Sun Yat-sen University, Guangzhou, China

**Keywords:** oral squamous cell carcinoma, gene signature, autophagy-related genes, nomogram, prognosis, The Cancer Genome Atlas

## Abstract

**Background:**

Autophagy, a highly conserved self-digesting process, has been deeply involved in the development and progression of oral squamous cell carcinoma (OSCC). However, the prognostic value of autophagy-related genes (ARGs) for OSCC still remains unclear. Our study set out to develop a multigene expression signature based on ARGs for individualized prognosis assessment in OSCC patients.

**Methods:**

Based on The Cancer Genome Atlas (TCGA) database, we identified prognosis-related ARGs through univariate COX regression analysis. Then we performed the least absolute shrinkage and selection operator (LASSO) regression analysis to identify an optimal autophagy-related multigene signature with the subsequent validation in testing set, GSE41613 and GSE42743 datasets.

**Results:**

We identified 36 prognosis-related ARGs for OSCC. Subsequently, the multigene signature based on 13 prognostic ARGs was constructed and successfully divided OSCC patients into low and high-risk groups with significantly different overall survival in TCGA training set (*p* < 0.0001). The autophagy signature remained as an independent prognostic factor for OSCC in univariate and multivariate Cox regression analyses. The area under the curve (AUC) values of the receiver operating characteristic (ROC) curves for 1, 3, and 5-year survival were 0.758, 0.810, 0.798, respectively. Then the gene signature was validated in TCGA testing set, GSE41613 and GSE42743 datasets. Moreover, Gene Ontology (GO), Kyoto Encyclopedia of Genes and Genomes (KEGG) analysis, and single-sample gene set enrichment analysis (ssGSEA) revealed the underlying biological characteristics and signaling pathways associated with this signature in OSCC. Finally, we constructed a nomogram by combining the gene signature with multiple clinical parameters (age, gender, TNM-stage, tobacco, and alcohol history). The concordance index (C-index) and calibration plots demonstrated favorable predictive performance of our nomogram.

**Conclusion:**

In summary, we identified and verified a 13-ARGs prognostic signature and nomogram, which provide individualized prognosis evaluation and show insight for potential therapeutic targets for OSCC.

## Introduction

Oral cancer is among the top 15 most prevalent cancers worldwide, with 354,864 new diagnoses and approximately 177,384 new death in 2018 (1). Generally, oral cancer is an extensive category of localization for a neoplasm arising in anterior two-thirds of the tongue, hard palate, gum, floor of the mouth, buccal mucosa, vestibule of the mouth, or retromolar area (1). Among these cancers, oral squamous cell carcinoma (OSCC) is the most common type, accounting for about 95% of all oral cancers (2). The most significant risk factors for OSCC are tobacco smoking, alcohol consumption, and areca nut chewing (3–5). Additionally, viruses and other microbes have been highly relevant to an increased risk of OSCC development, including persistent infections by human papilloma virus (HPV), Epstein-Barr virus (EBV), or *Candida albicans* (6–8). HPV types 16 and 18 are the most dominant types in HPV-positive oral cancers and were detected in approximately 24.4% of all oral cancers (6, 9). Despite remarkable advances in diagnosis and treatment for OSCC, the 5-year survival rate of patients with OSCC remains only 40–50% (10). Clinically, age, TNM stages, histological grades, tobacco, and alcohol consumption are used to assess the prognosis of OSCC patients (11). Nonetheless, these clinicopathological factors do not provide accurate information to predict a patient’s survival. In recent years, extensive efforts have been devoted to finding molecular prognostic biomarkers, although no effective biomarker has already been identified and clinically validated (12–14). Therefore, it is an urgent need to establish reliable prognostic biomarkers to help clinicians optimize and personalize treatment strategies.

Autophagy is a multi-step process by which damaged cellular components are transferred to lysosomes for degradation, resulting in nutrient cycling and metabolic adaptation (15). Therefore, autophagy plays an essential role in diverse biological and pathological processes, and its dysfunction may induce numerous diseases, including cancer, neurodegeneration disease, and infection (16). However, autophagy is a double-edged sword in tumorigenesis, with opposing, context-dependent roles in tumor formation and progression (17). For example, autophagy can eliminate impaired organelles and proteins to alleviate cellular damage and ensure metabolic stability, thereby inhibiting carcinogenesis in the early phase (18). Nevertheless, once the cancer has established, autophagy enhances tumor cell survival under stressful environments, and thus exerts a tumor-promoting effect (19).

A remarkable number of studies have demonstrated the relationship between autophagy and OSCC. For instance, a recent study indicated that autophagy helped maintain the stemness and promoted drug tolerance in OSCC (20). Another study reported that PIK3CA gene was frequently mutated in OSCC, which could lead to the activation of PI3K and downstream effectors [including mammalian target of rapamycin (mTOR)], and thus facilitated autophagy (21). Moreover, higher levels of LC3-II, which suggest increased basal levels of autophagy, have been revealed to be closely linked to unfavorable prognosis in OSCC (22). These research findings confirmed the involvement of autophagy in OSCC and indicated that autophagy-related genes (ARGs) may demonstrate tremendous promise as prognostic markers and therapeutic targets in OSCC. However, previous studies have been concentrated on the relationship between single or a few ARGs and OSCC prognosis. For instance, Liu PF *et al*. found the co-expression of higher MAP1LC3B and SQSTM1 was significantly associated with poor disease-specific survival and disease-free survival in OSCC patients (23). In addition, another tissue microarray analysis suggested that ATG4B, an autophagy related protease, could be a potential biomarker for diagnosis and prognosis for OSCC (24). To date, studies using large-scale expression patterns of ARGs to screen and develop molecular biomarkers and prognostic gene signature for OSCC are still lacking.

**Figure 1:**
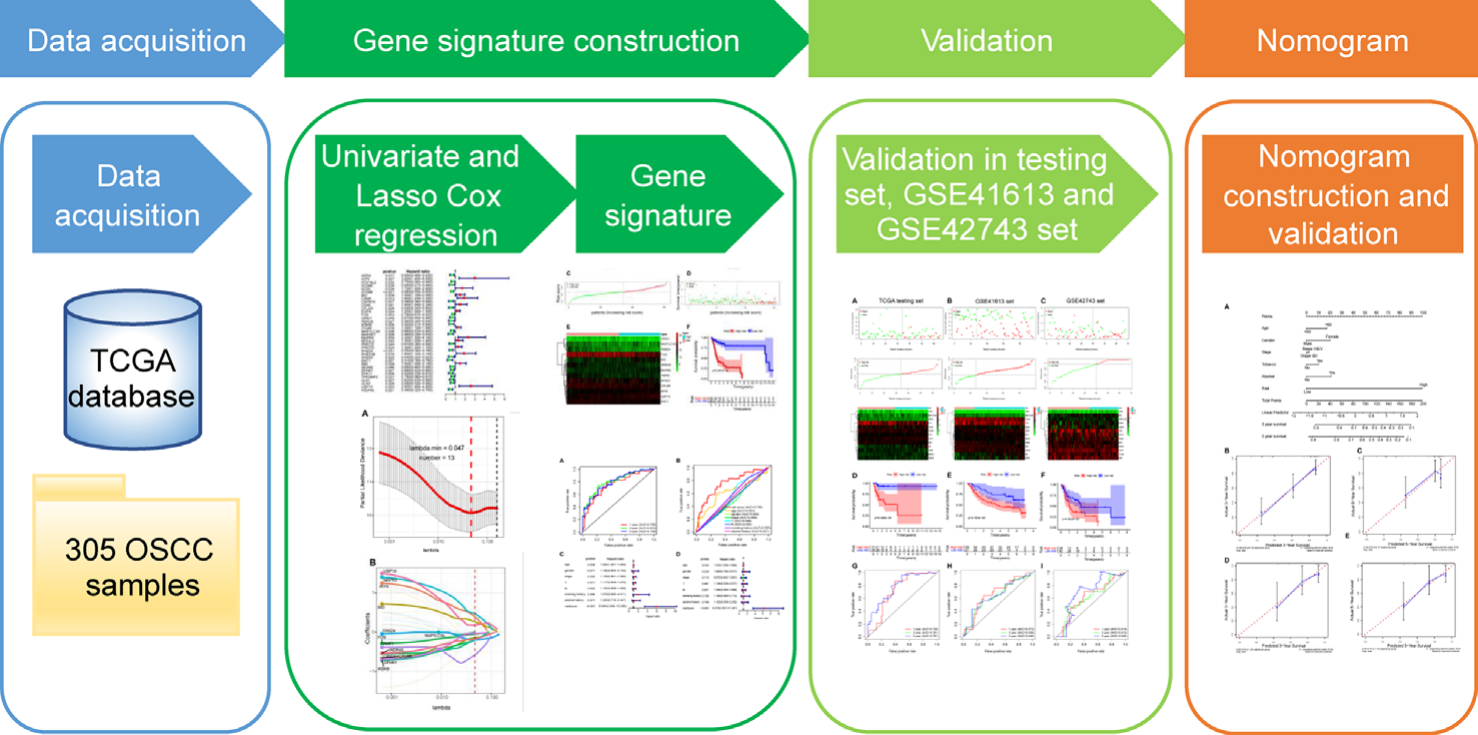
The workflow of the study for constructing the autophagy-related prognostic signature and nomogram in oral squamous cell carcinoma. TCGA, The Cancer Genome Atlas; OSCC, oral squamous cell carcinoma; LASSO, the least absolute shrinkage and selection operator.

Hence, this study set out to gain an in-depth understanding of the potential clinical utility of ARGs as prognostic biomarkers and to improve individualized prognosis assessment for OSCC patients. Firstly, we obtained ARGs from the Human Autophagy Database (HADb) and RNA-sequencing (RNA-seq) data of OSCC patients from The Cancer Genome Atlas (TCGA) database. Subsequently, we screened prognosis-related ARGs with Cox proportional hazard regression analysis. These acquired genes were then subjected to the least absolute shrinkage and selection operator (LASSO) Cox regression to identify an optimal autophagy-related multigene prognostic signature, followed by validation in both internal and external datasets. Finally, we integrated the gene signature and multiple clinical risk factors to construct a robust prognostic nomogram to enhance the accuracy of survival prediction for OSCC individuals. A workflow presenting our study design is illustrated in **Figure 1**.

## Materials and Methods

### Autophagy-Related Gene Set

A total of 222 ARGs were retrieved from the Human Autophagy Database (HADb, http://autophagy.lu/), a database providing a comprehensive and updated list of ARGs of human being.

### Data Acquisition of Oral Squamous Cell Carcinoma Datasets

All primitive RNA-seq datasets and clinical information of OSCC patients were downloaded and extracted from The Cancer Genome Atlas (TCGA, https://portal.gdc.cancer.gov/) and the Gene Expression Omnibus (GEO, https://www.ncbi.nlm.nih.gov/geo/) databases. In total, 305 OSCC samples were enrolled in our TCGA cohort. And then, TCGA-OSCC patients with complete clinical information were split into a training set (154 OSCC patients) and a testing set (internal validation set, 65 OSCC patients) randomly and homogeneously. Finally, GSE41613 (external validation set1, 96 OSCC patients) and GSE42743 (external validation set2, 68 OSCC patients) datasets were obtained from the GEO database for the validation studies.

### Construction and Validation of the Autophagy-Related Genes-Based Prognostic Signature

Univariate Cox proportional hazard regression analysis was performed to identify the ARGs significantly correlated with the overall survival (OS) in TCGA-OSCC cohort, using the survival package. These identified prognosis-related genes were used for subsequent construction of multigene signature. Then, we conducted LASSO Cox regression analysis to establish a prognostic multigene signature in training set with the pool of candidate prognosis-related ARGs. The aforementioned procedures were implemented with the glmnet package. According to the expression values of each sample, LASSO chooses the qualified prognostic genes to further calculate the risk score and assigns corresponding coefficient to each prognostic gene.

The risk score of each patient was calculated with the following formula: *risk score = expression level of Gene_1_ × β_1_ + expression level of Gene_2_ × β_2_ +…+ expression level of Gene_n_ × β_n_*, with β indicating the regression coefficient (25, 26). The optimal cutoff value of the risk score was identified using time-dependent receiver operating characteristic (ROC) analysis with survival ROC package. Afterwards, all OSCC patients in training set were separated into high-risk (with high risk score) and low-risk (with low risk score) groups by the cutoff point. Survival differences between the two groups were evaluated by Kaplan-Meier (K-M) survival curve and compared by log-rank test. In order to assess the predictive accuracy of the prognostic signature, time-dependent ROC curves for 1, 3, and 5-year survival were constructed and the area under the curve (AUC) values were calculated with the survival ROC package. Furthermore, univariate and multivariate Cox regression analyses for OS were applied to compare the prognostic relevance between the autophagy signature and other clinical factors (including age, gender, stage, T-stage, N-stage, smoking, and alcohol history) in TCGA training set. Finally, we used TCGA testing set as well as two GEO cohorts (GSE41613 and GSE42743 sets) as internal and external validation sets to examine the universality and reliability of the gene signature by a similar approach. The same formula and the same cutoff value were used in the risk score calculation and subsequent groups division in the three validation sets.

### Identification of the Prognostic Signature Associated Biological Characteristics and Signaling Pathways

All the prognostic genes involved in our signature were subjected to GO and Kyoto Encyclopedia of Genes and Genomes (KEGG) analysis to explore the associated biological characteristics and potential signaling pathways. Next, we used these genes to establish a protein-protein interaction (PPI) network by the STRING database (https://string-db.org/) and the PPI network was then visualized through Cytoscape (Version 3.6.1) software. Moreover, single-sample gene set enrichment analysis (ssGSEA) was conducted to uncover the differentially enriched signaling pathways between low and high-risk groups. First, GSVA package and its ssGSEA method were used to calculate the enrichment score of each sample on different gene sets. Then significantly differentially enriched gene sets between low and high-risk groups were identified using the limma package and the top 30 of these gene sets were displayed by heat map. Moreover, we performed Pearson’s correlation analysis using the corrplot package in order to confirm the correlation between these pathways and the risk score.

### Construction and Validation of Nomogram

To estimate the probability of 3- and 5-year survival for OSCC patients, we used age, gender, stage, tobacco history, alcohol history, and risk score to construct a nomogram in training set, *via* the rms package. Next, calibration curves were plotted to graphically evaluate the consistency between actual and predicted survival (27). Additionally, we quantitatively evaluated the prediction performance of the nomogram by the concordance index (C-index). Finally, the prognostic nomogram was internally verified in TCGA testing set in the same way.

### Statistical Analysis

All statistical analyses were conducted using the R software (version 3.6.2). Two-sided t test was used to check the relationship between the risk score and clinical features. Survival curves were plotted by the K-M method and assessed with log-rank test. Univariate and multivariate analyses were performed by the Cox proportional hazard regression analysis. During all the statistical tests, a *p*-value of less than 0.05 was considered as statistically significant difference.

## Results

### Construction of the Autophagy-Related Genes-Based Prognostic Signature

**Figure 2:**
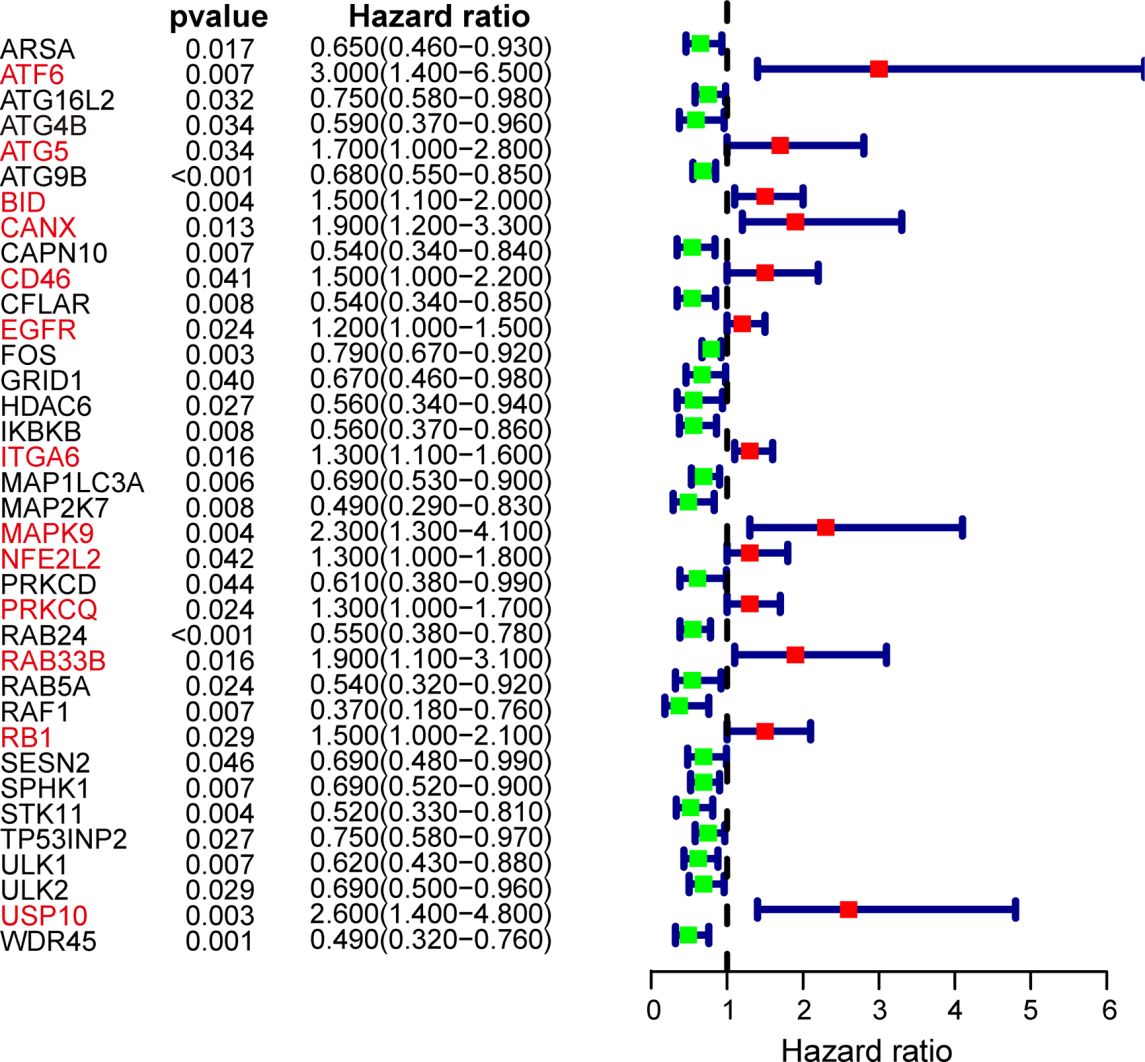
Forest plot of autophagy-related genes (ARGs) associated with OSCC survival. Genes in red font represent risk genes and in black font represent protective genes. Hazard ratios and 95% confidence intervals were calculated using univariate Cox regression analysis.

After excluding patients followed up less than 1 month, the remaining OSCC patients in TCGA cohort were subjected to univariate Cox proportional hazard regression analysis to explore the prognostic value of ARGs. Considering the criteria for *p* < 0.05, a total of 36 ARGs were screened out as prognosis-related genes. The forest map showed the hazard ratio and the corresponding confidence interval of each prognosis-related ARG, which revealed that most of these genes were protective genes (**Figure 2**). Next, these prognostic genes were included in further LASSO Cox regression analysis.

**Figure 3:**
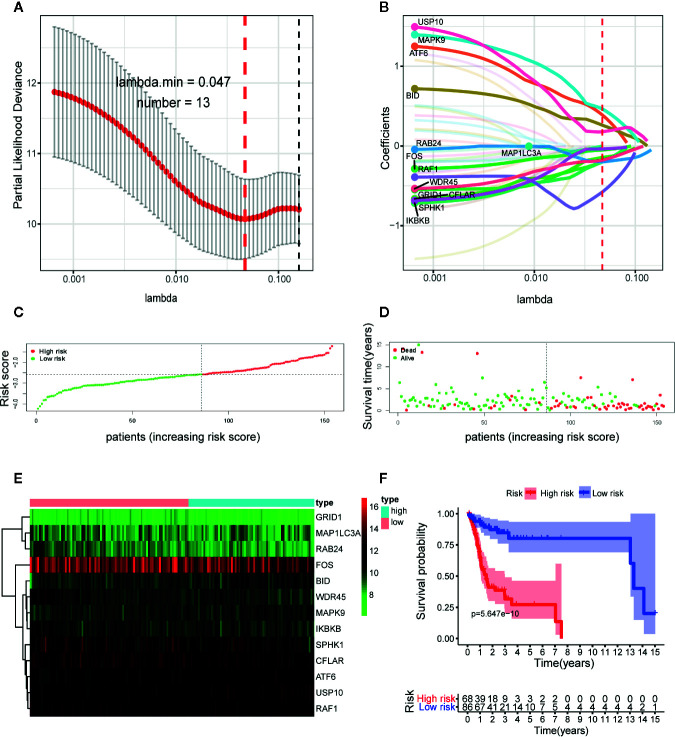
Construction of the autophagy-related gene signature in The Cancer Genome Atlas (TCGA) training set by the least absolute shrinkage and selection operator (LASSO) regression analysis for predicting OSCC patients’ overall survival. **(A)** Selection of the optimal parameter (lambda) in the LASSO model. **(B)** LASSO coefficient profiles of the 13 prognostic autophagy-related genes (ARGs). A coefficient profile plot was generated against the log (lambda) sequence. Distribution of risk score **(C)** and life status, survival time **(D)** of patients in training set. The risk scores are arranged in ascending order from left to right and each dot indicates an individual in training set. The black dotted line is the optimum cutoff dividing patients into low and high-risk groups. **(E)** Heat map of the expression profile of the included ARGs. The colors from green to red indicate the expression level from low to high. **(F)** Kaplan-Meier analysis of OSCC patients stratified by the cut-off risk score value. *p*-value was calculated by log-rank test.

In subsequent model construction, we eliminated samples in which key clinical information (TNM stage) was missing and then used caret package to divide the TCGA-OSCC cohort into a training set and a testing set homogeneously and randomly. The information of the two TCGA sets and two GEO validation sets was detailed in **Table 1**. By performing LASSO Cox regression analysis in TCGA training set, the prognostic ARGs contributing most to the OS of OSCC were generated and the corresponding regression coefficients were computed. **Figure 3A** illustrated that the model achieved the best performance while 13 genes were involved. **Figure 3B** indicated the LASSO regression coefficient profiles of the 13 genes. The full name and regression coefficient of each gene were summarized in **Table 2**. Among the 13 prognostic genes, four genes (USP10, ATF6, MAPK9, BID) were considered as risk genes (HR > 1), while the other genes (FOS, MAP1LC3A, SPHK1, GRID1, IKBKB, RAB24, CFLAR, WDR45, RAF1) were considered as protective genes (HR < 1).

**Table 1:** Demographics and clinical characteristics of oral squamous cell carcinoma (OSCC) patients in The Cancer Genome Atlas (TCGA) training set, testing set, and two external validation sets.

Variables	Training setn = 154	Testing setn = 65	GSE41613 setn = 96	GSE42743 setn = 68
Gender				
Male	108	42	65	53
Female	46	23	31	15
Age				
<60	66	24	50	34
≥60	88	41	46	34
TNM stage				
Stage I–II	43	18	41	NA
Stage III–IV	111	47	55	NA
T (tumor)				
T1–T2	61	23	NA	27
T3–T4	93	42	NA	41
N (lymph node)				
N0	80	40	NA	39
N1–N3	74	25	NA	29
M (metastasis)				
M0	153	65	NA	NA
M1	1	0	NA	NA
Smoking history				
Yes	113	42	NA	54
No	37	22	NA	14
Unknown	4	1	NA	0
Alcohol history				
Yes	101	43	NA	NA
No	50	20	NA	NA
Unknown	3	2	NA	NA

NA, not available.

**Table 2:** List of 13 autophagy-related genes (ARGs) in the prognostic gene signature.

Gene symbol	Full name	Lasso coefficient
USP10	Ubiquitin specific peptidase 10	0.1776
ATF6	Activating transcription factor 6	0.3891
MAPK9	Mitogen-activated protein kinase 9	0.4648
BID	BH3 interacting domain death agonist	0.2603
FOS	FBJ murine osteosarcoma viral oncogene homolog	−0.0506
MAP1LC3A	Microtubule-associated protein 1 light chain 3 alpha	−0.0006
SPHK1	Sphingosine kinase 1	−0.0363
GRID1	Glutamate receptor, ionotropic, delta 1	−0.2013
IKBKB	Inhibitor of kappa light polypeptide gene enhancer in B-cells, kinase beta	−0.1986
RAB24	RAB24, member RAS oncogene family	−0.2002
CFLAR	CASP8 and FADD-like apoptosis regulator	−0.1427
WDR45	WD repeat domain 45	−0.1799
RAF1	v-raf-1 murine leukemia viral oncogene homolog 1	−0.5773

Next, we constructed the ARGs-based prognostic signature with the following formula: risk score = (0.1776 × expression value of USP10) + (0.3891 × expression value of ATF6) + (0.4648 × expression value of MAPK9) + (0.2603 × expression value of BID) + (−0.0506 × expression value of FOS) + (−0.0006 × expression value of MAP1LC3A) + (−0.0363 × expression value of SPHK1) + (−0.2013 × expression value of GRID1) + (−0.1986 × expression value of IKBKB) + (−0.2002 × expression value of RAB24) + (−0.1427 × expression value of CFLAR) + (−0.1799 × expression value of WDR45) + (−0.5773 × expression value of RAF1). Based on the formula, we calculated the risk score of each patient in training set. The optimal cutoff value (−2.5842) of the risk score was determined using ROC analysis. Then all patients in training set were split into a high-risk group (n = 68) and a low-risk group (n = 86) with the cutoff point. **Figure 3** displayed the distribution of risk score (**Figure 3C**), survival status (**Figure 3D**) as well as the expression patterns of the 13 ARGs involved in our signature (**Figure 3E**) in low and high-risk groups of training set. As illustrated, patients of high-risk group had a lower probability to survive and had a tendency to express risk genes while conflicting results were observed in low-risk group.

**Figure 4:**
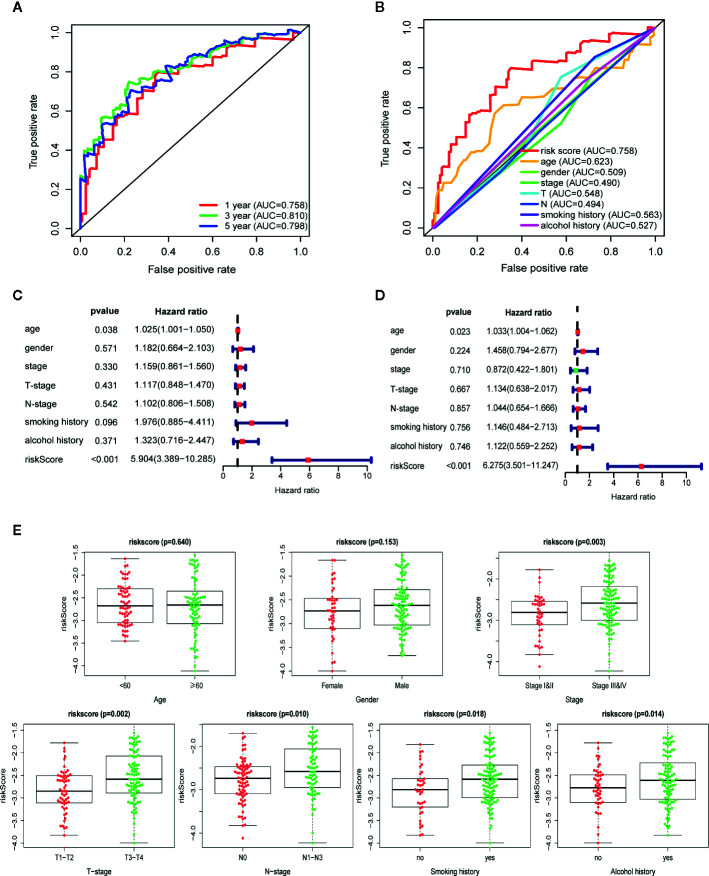
Autophagy-related gene signature shows good predictive performance in The Cancer Genome Atlas (TCGA) training set. **(A)** The receiver operating characteristic (ROC) curves of the prognostic signature for 1-, 3-, and 5-year survival. **(B)** ROC curves of the prognostic signature and clinical risk factors for 1-year survival. Forest plots of univariate **(C)** and multivariate **(D)** Cox regression analyses involving the risk score and clinical risk factors. **(E)** Clinical significance of the prognostic signature in TCGA training set. Risk score in different age, gender, tumor stage, T-stage, N-stage, smoking history, and alcohol history. *p*-values were calculated by two-sided t test.

To further assess the predictive performance of the prognostic signature in OSCC patients, we performed K-M survival analysis and time-dependent ROC analysis in training set. K-M survival analysis demonstrated that patients in high-risk group had a significantly worse OS compared to those in low-risk group with a *p* = 5.647 × 10^−10^ in the log-rank test (**Figure 3F**). In addition, as shown in **Figure 4A**, the AUC values of the ROC curves for 1, 3, and 5-year survival were 0.758, 0.810, 0.798, respectively, which indicated a favorable predictive accuracy of the prognostic signature. To compare the survival predictive power of our gene signature with clinical parameters, we constructed another ROC analysis involving several clinical parameters for 1-year OS. **Figure 4B** demonstrated a superior prediction performance of our gene signature with an AUC value of 0.758, compared to age (AUC = 0.623), gender (AUC = 0.509), stage (AUC = 0.490), T-stage (AUC = 0.548), N-stage (AUC = 0.494), smoking history (AUC = 0.563), and alcohol history (AUC = 0.527).

Following that, we performed univariate and multivariate Cox regression analyses in training set to explore if the autophagy signature could be an independent prognostic factor for OSCC. Univariate analysis indicated that the risk score (HR = 5.904, 95% CI = 3.389−10.285, *p* < 0.001) and age (HR = 1.025, 95% CI = 1.001−1.050, *p *= 0.038) were significantly correlated with the prognosis of OSCC patients (**Figure 4C**). Moreover, the risk score remained as an independent predictor for OSCC patients in multivariate analysis (HR = 6.275, 95% CI = 3.501−11.247, *p* < 0.001), after adjusting for clinical features including age, gender, stage, T-stage, N-stage, smoking, and alcohol history (**Figure 4D**).

The relationship between the ARGs-based risk score and several clinical parameters was further analyzed. Results illustrated that the risk score was significantly higher in TNM-stage III–IV than in I–II (*p* = 0.003), higher in T-stage T3–T4 than in T1–T2 (*p* = 0.002), higher in N-stage N1–N3 than in N0 (*p* = 0.010), higher in patients with smoking history than non-smokers (*p *= 0.018) and higher in patients with alcohol history than non-drinkers (*p* = 0.014). In addition, no statistically significant correlation was found between the risk score and age (*p* = 0.640) as well as gender (*p* = 0.153) (**Figure 4E**).

### Evaluation of the Autophagy-Related Genes-Based Prognostic Signature in the Internal and External Validation Cohorts

**Figure 5:**
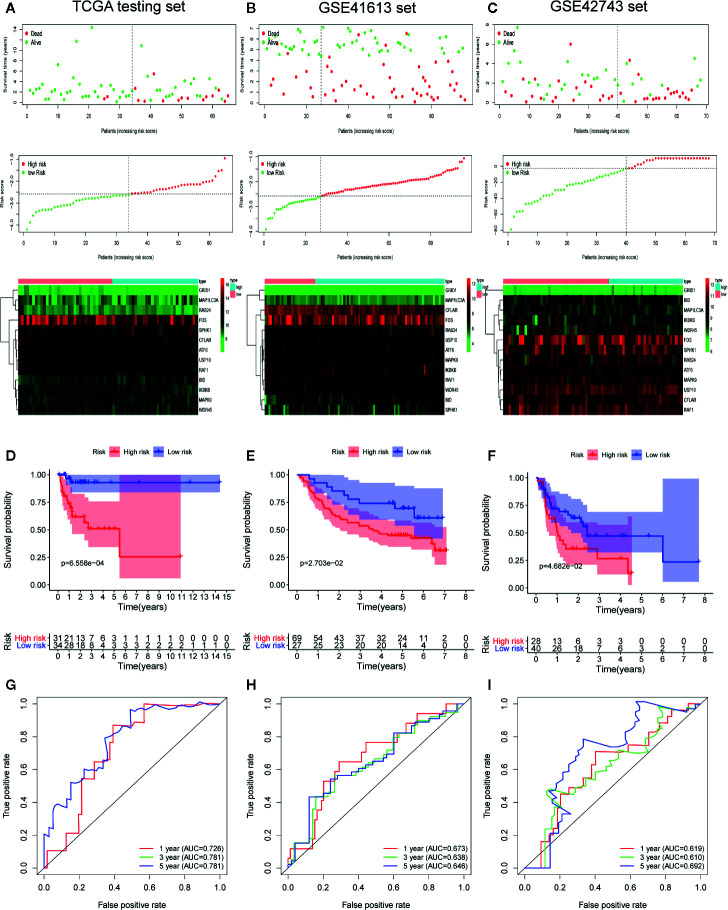
Internal and external validation of the prognostic value of the autophagy-related gene signature in The Cancer Genome Atlas (TCGA) testing set, GSE41613 set and GSE42743 set. **(A–C)** The distribution of survival time, life status, risk score, and the prognostic 13-ARGs expression patterns in testing set **(A)**, GSE41613 **(B)**, and GSE42743 set **(C)**. The risk scores are arranged in ascending order from left to right and each dot indicates an oral squamous cell carcinoma (OSCC) individual. The black dotted line is the optimum cutoff dividing patients into low and high-risk groups. The colors from green to red in heatmap indicate the expression level from low to high. **(D–F)** Kaplan-Meier plots compare overall survival between patients in low and high-risk groups in testing set **(D)**, GSE41613 **(E)**, and GSE42743 set **(F)**. **(G–I)** The receiver operating characteristic (ROC) curves of the prognostic signature for 1-, 3-, and 5-year survival in testing set **(G)**, GSE41613 **(H)**, and GSE42743 set **(I)**. Only two curves are plotted in panel **(G)** owing to the coincidence of ROC curves for 3- and 5-year survival. *p*-values were calculated by log-rank test.

To confirm the strong predictive potential of the prognostic signature in different datasets, we used the same formula to compute the risk score for each patient in TCGA testing set (internal validation set), GSE41613 (external validation set1), and GSE42743 sets (external validation set2). In each cohort, patients were separated into low and high-risk groups based on the same cut off value. **Figure 5** displayed the distribution of risk score, survival status as well as the expression patterns of the 13 ARGs involved in our signature in low and high-risk groups of testing set (**Figure 5A**), GSE41613 set (**Figure 5B**) and GSE42743 set (**Figure 5C**). Similar results were observed in all the validation sets that patients of high-risk group had a lower probability to survive and had a tendency to express risk genes while the low-risk group showed conflicting results.

Furthermore, we performed K-M survival analysis and time-dependent ROC analysis to accurately assess the predictive performance of the prognostic signature in different datasets. As illustrated in **Figure 5D**, a total of 65 OSCC patients in testing set were separated into a high-risk group (n = 31) and a low-risk group (n = 34), and patients in high-risk group had a significantly worse OS compared to those in low-risk group (log-rank *p* = 6.558 × 10^−4^). Following that, the prognostic signature also demonstrated a favorable predictive power for 1-, 3-, and 5-year survival in testing set, with AUC values of 0.726, 0.781, and 0.781, respectively (**Figure 5G**). Additionally, a total of 96 patients in GSE41613 were divided into a high-risk group (n = 69) and a low-risk group (n = 27) (**Figure 5E**). A total of 68 patients in GSE42743 were also divided into a high-risk group (n = 28) and a low-risk group (n = 40) (**Figure 5F**). The K-M curves indicated that the OS of patients in high-risk group was significantly worse than those in low-risk group in both two external validation sets (log-rank *p* = 2.703 × 10^−2^ for GSE41613 set and log-rank *p* = 4.682×10^−2^ for GSE42743 set). Moreover, the AUC values of the ROC curves for 1-, 3-, and 5-year survival in GSE41613 set were 0.673, 0.638, and 0.646, respectively (**Figure 5H**) and in GSE42743 set, the AUC values for 1-, 3-, and 5-year survival were 0.619, 0.610, and 0.692, respectively (**Figure 5I**). Collectively, our results proved that the 13-ARGs prognostic signature was predictive of survival in OSCC patients both in internal and external validation sets.

### Identification of the Prognostic Signature Associated Biological Characteristics and Signaling Pathways

**Figure 6:**
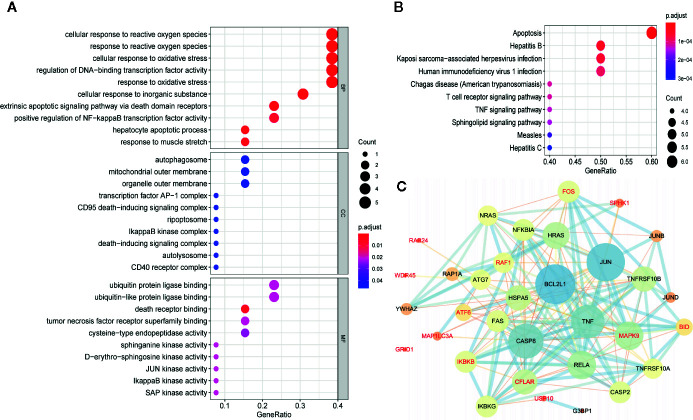
Gene functional enrichment analysis and protein-protein interaction (PPI) network of the 13 autophagy-related genes (ARGs) in the prognostic gene signature. **(A)** GO enrichment analysis of the 13 prognostic ARGs. The y‐axis stands for significantly enriched GO terms, and the x‐axis stands for the different gene ratio. **(B)** KEGG pathway enrichment analysis of the 13 prognostic ARGs. The y‐axis represents significantly enriched KEGG pathways, and the x‐axis represents different gene ratio. **(C)** Proteins interacted with the 13 prognostic ARGs (red font). A large node means a higher interaction degree and a thicker line indicates a stronger data support. BP, biological processes; CC, cellular components; MF, molecular functions.

**Figure 7:**
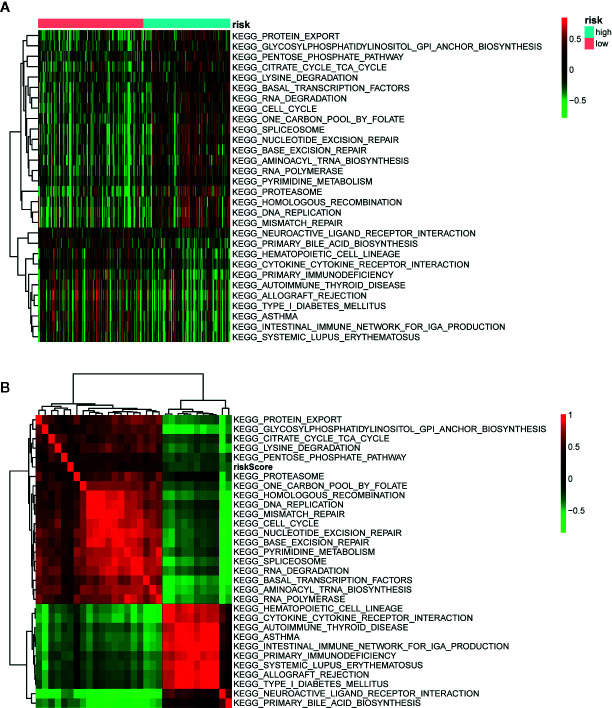
Pathway profiles and correlation analysis across The Cancer Genome Atlas (TCGA) training set. **(A)** Pathway profiles of training set. Rows and columns represent pathways and patients, respectively. Each cell represents an enrichment score of pathway activity calculated by single-sample gene-set enrichment analysis (ssGSEA) and the colors from green to red indicate the enrichment score from low to high. The red and blue bars stand for low and high-risk groups, respectively. **(B)** Pearson’s correlation analysis of the risk score and pathways. Each cell of the heatmap represents a correlation coefficient and the colors from green to red indicate the correlation coefficient from negative to positive.

To investigate the biological characteristics and potential signaling pathways related to the 13 prognostic ARGs, we performed GO and KEGG analysis. GO enrichment analysis revealed that these genes could be associated with several vital biological processes, including response to reactive oxygen species and oxidative stress, regulation of DNA−binding transcription factor activity and apoptotic signaling pathway (**Figure 6A**). KEGG analysis indicated that these prognostic genes were significantly enriched in apoptosis, hepatitis B, Kaposi sarcoma-associated herpesvirus infection, and human immunodeficiency virus 1 infection (**Figure 6B**). Moreover, to further investigate the biological role of the 13 genes in prognostic signature, we introduced additional 20 genes to generate a tight PPI network by STRING online database (**Figure 6C**). In addition, we performed ssGSEA analysis in TCGA training set to identify significantly differentially enriched gene sets between high and low-risk groups. As illustrated in **Figure 7A**, a series of pathways related to nutrition metabolism, RNA biosynthetic, cell cycle, DNA repair and replication were significantly upregulated in high-risk group. Meanwhile, some immune-related signaling pathways such as hematopoietic cell lineage, cytokine-cytokine receptor interaction, autoimmune thyroid disease and type I diabetes mellitus were significantly enriched in the low-risk group. Moreover, we performed Pearson’s correlation analysis to validate the correlation between these pathways and the risk score. **Figure 7B** highlighted the correlation that most of these pathways (such as pathways related to nutrition metabolism, RNA biosynthetic and splicing, cell cycle, DNA repair and replication) were positively related to the risk score while a small number of pathways (such as hematopoietic cell lineage, cytokine-cytokine receptor interaction, autoimmune thyroid disease, type I diabetes mellitus, and so on) presented a negative correlation. Overall, the prognostic signature mainly contributed to cancer and immune-related pathways, the dysregulation of which were closely associated with tumor progression.

### Construction and Validation of Nomogram

**Figure 8:**
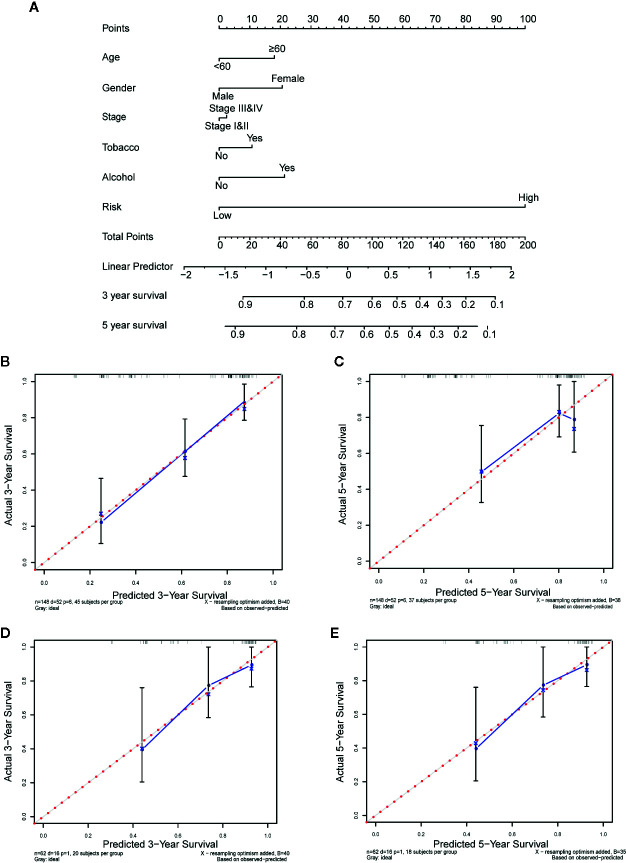
Nomogram to predict the 3-, and 5-year survival probability of oral squamous cell carcinoma (OSCC) patients. **(A)** Prognostic nomogram for predicting overall survival (OS) of OSCC patients based on The Cancer Genome Atlas (TCGA) training set. **(B, C)** The calibration plots for predicting 3-year **(B)** and 5-year survival **(C)** in training set. **(D, E)** The calibration plots for predicting 3-year **(D)** and 5-year survival **(E)** in testing set. Nomogram-predicted survival and actual survival were plotted on the x-axis and y-axis, respectively. The red dotted line represents the best prediction and the blue solid line represents the nomogram-prediction. The vertical bars represent a 95% confidence interval.

In order to quantitatively estimate survival probability for OSCC individuals in the clinical setting, we constructed a nomogram in TCGA training set which integrated both the autophagy signature and multiple clinical parameters (age, gender, TNM-stage, tobacco, and alcohol history) to predict 3- and 5-year OS probabilities. **Figure 8A** indicated that each factor was assigned specific points in proportion according to its contribution to survival and it was not surprising to see the risk score was the most crucial factor among the various parameters. A C-index, ranging from 0.5 to 1.0, was calculated to quantitatively estimate the prediction performance of our nomogram. The value of 0.5 and 1.0 indicates a random chance and a remarkable ability for survival prediction with the nomogram, respectively. The C-index of the nomogram was 0.737 (95% CI = 0.658−0.816, *p *= 4.489×10^−9^). In addition, we constructed calibration curves to graphically evaluate the consistency between nomogram predicted and actual survival. As shown in **Figure 8**, actual and predicted survival matched very well in terms of 3-year (**Figure 8B**) and 5-year (**Figure 8C**) OS in training set. Next, the nomogram was validated in TCGA testing set. GSE41631 and GSE42743 sets were not included as validation sets for the nomogram owing to the incomplete clinical information. In testing set, the C-index of the nomogram was 0.706 (95% CI = 0.583−0.829, *p* = 1.059 × 10^−3^) and the calibration plots also demonstrated strong consistency between the nomogram prediction and actual observation for 3-year (**Figure 8D**) and 5-year (**Figure 8E**) OS. Overall, these findings confirmed the prediction reliability for survival probability of our nomogram in OSCC patients.

## Discussion

OSCC is a progressive disease with heterogeneous prognosis and high mortality rate, and thus accurate prognostic biomarkers are urgently needed to improve prognosis assessment and individualized treatment (1, 12). Although previous studies have investigated numerous molecular biomarkers and multiple gene expression signatures for OSCC, a comprehensive analysis of global expression patterns of ARGs has not been conducted yet (12, 28–30). In this study, we mined the existing RNA-seq expression data of OSCC to screen prognosis-related ARGs and establish a multigene expression signature from the view of autophagy for the first time. Our results show that an ARGs-based prognostic signature can be applied for prognosis stratification in OSCC patients, which will contribute to individualized treatment and shed new light on autophagy targeting therapies.

In this study, we extracted the mRNA expression data of 222 ARGs in TCGA-OSCC cohort and developed a 13-ARGs prognostic signature using LASSO regression analysis. K-M curves indicated that the gene signature could successfully divide OSCC patients into low and high-risk groups with significantly different OS. Univariate and multivariate Cox regression analyses revealed that the gene signature could be an independent prognostic factor for OSCC. The ROC analysis also indicated a better prediction performance of our gene signature compared with other clinical risk factors. Additionally, the prognostic power of our gene signature could be validated in independent GEO datasets and internal testing set, indicating favorable universality and reliability. In order to achieve better predictive performance, we combined the gene signature with multiple clinical risk factors (age, gender, TNM-stage, tobacco, and alcohol history) to establish a nomogram to quantitatively estimate survival probability for OSCC. Calibration plots demonstrated favorable consistency between actual and predicted survival both in training set and testing set. Thus, our gene signature and nomogram may provide an accurate and reliable prediction approach for the prognosis of OSCC patients and help clinicians optimize and personalize treatment strategies.

Among these 13 ARGs in our prognostic gene signature, most of them have been reported to be closely associated with the development and prognosis of OSCC or other malignancies. MAP1LC3A, WDR45, and RAB24 are vital components of the autophagy machinery. Prior study has noted that MAP1LC3A expression was suppressed in many tumor cell lines, which suggested that it might be highly linked to the tumorigenesis of gastric cancer, esophageal squamous carcinoma, and osteosarcoma (31). Another immunohistochemistry study reported that the expression of MAP1LC3A at the surgical margins in OSCC patients could be a biomarker indicating local recurrence as well as poor prognosis (32). A cross-cancer profiling of ARGs alterations has found that WDR45 was under significantly somatic mutations in endometrial carcinoma, suggesting WDR45 mutation could play a positive role in tumorigenesis (33). Furthermore, RAB24 protein was reported to promote malignant phenotype transformation in hepatocellular carcinoma (34). BID is a death-inducing member of the BCL-2 family and previous studies indicated that BID could be a prognostic indicator for OSCC (35, 36). CFLAR encodes FLIP, a multifunctional protein involved in various cellular processes including apoptosis, necroptosis, and autophagy (37). A number of studies have shown that FLIP could be a potential prognostic biomarker and therapeutic target in non‐small cell lung cancer (38). IKBKB and SPHK1 encode kinase regulating the activation of NF-κB pathway and other signaling pathways. Several studies have demonstrated a tumor-promoting effect of IKK (encoded by IKBKB) in intestinal, lung, and pancreatic cancer (39–41). Over expression of SPHK1 has been reported to be associated with invasiveness, migration and poor prognosis of OSCC (42, 43). FOS and RAF1 are proto-oncogenes closely related to cell proliferation, differentiation, migration, and transformation. Studies have found that differential expression and activation of FOS and RAF1 were deeply involved in human oral carcinogenesis (44, 45). FOS has been reported to promote cell invasion and migration by CD44 pathway in OSCC, suggesting its potential value as a prognostic marker, especially in lymph node metastasis (46). MAPK9 is a member of the MAP kinase family. A recent study has shown that MAPK9 activity played an indispensable part in invasiveness and BRAFi resistance of melanoma cell (47). As for USP10, decreased expression of USP10 has been proved to be an indicator of poor prognosis in lung cancer and epithelial ovarian cancer (48, 49). Moreover, numerous studies have demonstrated that USP10 inhibited mTOR activation and promoted oncogene-induced senescence to exert a tumor-suppressive effect (50, 51). ATF6 plays a significant part in activating the unfolded protein response (UPR) during endoplasmic reticulum stress, which has been reported to be promotive in cancer tumorigenesis and metastasis (52). In addition, others have shown that ATF6 was related to reduced time of disease-free survival in colorectal cancer (53). Unger K *et al*. found upregulation of GRID1 in grade 3 and low-grade lymph-node-positive breast cancers and rearrangement of GRID1 in numerous breast cancers, indicating its potential value as tumor marker (54). In summary, all these prognostic ARGs in the gene signature are strongly associated with cancers mainly by regulating autophagy and apoptosis. Therefore, it is reasonable to assume that these ARGs can be used as prognostic biomarkers and potential therapeutic targets for OSCC.

To better understand the biological role and signaling pathways of the 13 ARGs in the signature, we performed GO, KEGG, ssGSEA analysis, and PPI network. GO and KEGG analysis uncovered that these prognostic genes were significantly associated with response to oxidative stress and apoptosis signaling pathway. Consistently, ssGSEA analysis indicated that cell cycle, DNA repair, and DNA replication pathways were significantly enriched in high-risk group, all of which were well-known cancer-related pathways. Moreover, our results validated the correlation between the risk score and these pathways. PPI network showed the interaction network of the 13 ARGs and additional 20 genes closely linked to the signature. Top three genes with highest connectivity degree in the PPI network were BCL2L1 (degree = 23), JUN (degree = 22), and CASP8 (degree = 20). Caspase8 (encoded by CASP8), the activation of which can be suppressed by FLIP (encoded by CFLAR), plays a crucial part in the execution-phase of death receptor‐mediated extrinsic apoptotic pathway and TNFα‐induced apoptotic and necroptotic cell death (55, 56). Additionally, caspase8 regulates the activation of the pro-apoptotic Bcl-2 family member Bid (encoded by BID) to affect intrinsic mitochondrial-mediated apoptotic pathway, which can be suppressed by apoptosis inhibitor Bcl-XL (encoded by BCL2L1) as well as FLIP (56). Moreover, c-Fos and USP family indirectly affects the activation of caspase8 by regulating the transcription and ubiquitination of FLIP (57, 58). Jun proteins (c-Jun, JunB, JunD) compose AP-1 transcription factor with Fos proteins (c-Fos, FosB, Fra-1, and Fra2) and other activating transcription factor protein families (59). Similar to autophagy, AP-1 has been reported to play a dual role in oncogenesis (60, 61). Several studies indicated that increased activity of AP-1 can exert both pro-apoptotic and anti-apoptotic effects in human tumor cells (62, 63). In summary, we speculate that the interplay between autophagy and apoptosis plays a vital role in the prognosis of OSCC patients and our results provide some insight into the underlying molecular mechanisms of tumor progression in OSCC.

With the rapid development of high-throughput sequencing technology and bioinformatics, a large number of gene signatures based on various kinds of RNA expression data have been constructed to predict prognosis in OSCC (28, 29). Nevertheless, compared with our study, these studies lacked independent validation in external datasets. Furthermore, most of these studies only concentrated on molecular biomarkers and ignored the value of traditional clinical parameters. We integrated clinical parameters with the autophagy-related signature for predicting survival in OSCC for the first time, which showed great promise for clinical application. However, there are still some limitations in our study. First, our study is retrospective thus the gene signature and nomogram need to be further verified in prospective studies and multi-center clinical trials. Second, the information from TCGA and GEO databases is limited and incomplete. Several potential risk factors such as radiotherapy and pathological features were not enrolled in our nomogram. Third, more investigations are needed to further reveal the function and mechanisms of these prognostic ARGs in OSCC.

In conclusion, our study identified a 13-ARGs prognostic signature based on a thorough analysis of ARGs expression profile in OSCC. Then we constructed a promising prognostic nomogram by integrating both the gene signature and multiple clinical parameters. Our gene signature and nomogram may provide an accurate and reliable prediction approach for the prognosis of OSCC patients and thus help clinicians optimize and personalize treatment strategies. The genes identified in the prognostic signature also provide some insight for novel prognostic biomarkers and potential therapeutic targets. However, large-scale and prospective clinical investigations should be carried out to validate the clinical utility of our signature and associated underlying biological mechanisms remain to be further unveiled.

## Data Availability Statement

Publicly available datasets were analyzed in this study. This data can be found here: OSCC cohort from TCGA database, https://portal.gdc.cancer.gov/; GSE41613 and GSE42743 datasets from GEO database, https://www.ncbi.nlm.nih.gov/geo.

## Author Contributions

CH and HC conceived and designed the study. CH, YZ, and FS performed the data analysis. CH, HC, YZ, and SH analyzed and interpreted the results. CH wrote the original manuscript. HC and JH reviewed and revised the manuscript. All authors contributed to the article and approved the submitted version.

## Funding

This research was supported by the National Natural Science Foundation of China [grant numbers 81874128]; Sun Yat-Sen University Clinical Research 5010 Program [grant numbers 2015018].

## Conflict of Interest

The authors declare that the research was conducted in the absence of any commercial or financial relationships that could be construed as a potential conflict of interest.

The handling editor declared a shared affiliation with the authors at time of review.
